# Development of a High-throughput Agar Colony Formation Assay to Identify Drug Candidates against Medulloblastoma

**DOI:** 10.3390/ph13110368

**Published:** 2020-11-05

**Authors:** Mohammed Sedeeq, Ahmed Maklad, Nuri Gueven, Iman Azimi

**Affiliations:** School of Pharmacy and Pharmacology, College of Health and Medicine, University of Tasmania, Hobart TAS 7005, Tasmania, Australia; mohammed.sedeeq@utas.edu.au (M.S.); ahmed.maklad@utas.edu.au (A.M.); nuri.guven@utas.edu.au (N.G.)

**Keywords:** assay development, high-throughput, drug discovery, calcium signalling, medulloblastoma

## Abstract

Medulloblastoma (MB) is the most common malignant childhood brain cancer. High-risk MB tumours have a high incidence of metastasis and result in poor patient survival. Drug screens, commonly used to identify potential novel therapeutic agents against MB, focus on 2D cell proliferation and viability assays given that these assays are easily adaptable to high-throughput regimes. However, 2D models fail to address invasive characteristics that are crucial to MB metastasis and are thus not representative of tumour growth in vivo. In this study, we developed a 3D 384-well agar colony formation assay using MB cells of molecular subgroup 3 that is associated with the highest level of metastasis. Two fluorescence substrates, resazurin and glycyl-phenylalanyl-aminofluorocoumarin (GF-AFC) that measure cell viability via distinct mechanisms were used to assess the growth of MB cells in the agar matrix. The assay was optimised for seeding density, growth period, substrate incubation time and homogeneity of the fluorescent signals within individual wells. Our data demonstrate the feasibility to multiplex the two fluorescent substrates without detectable signal interference. This assay was validated by assessing the concentration-dependent effect of two commonly used chemotherapeutic agents clinically used for MB treatment, vincristine and lomustine. Subsequently, a panel of plasma membrane calcium channel modulators was screened for their effect on the 3D growth of D341 MB cells, which identified modulators of T-type voltage gated and ORAI calcium channels as selective growth modulators. Overall, this 3D assay provides a reproducible, time and cost-effective assay for high-throughput screening to identify potential drugs against MB.

## 1. Introduction

Medulloblastoma (MB), the most common paediatric malignant brain tumour, represents 20% of all childhood brain cancers [1]. MB is highly aggressive and often associated with high mortality rates. In children, the tumour usually arises in the posterior fossa region of the brain as densely packed cells with high invasive ability allowing them to spread to other areas of the central nervous system [1].

Current treatment strategies for MB involve surgical resection, radiotherapy and chemotherapy as the most effective form of therapy available. Although these treatment modalities improve survival rate to 60%, it is associated with severe complications. The cytotoxic effects of these therapies have a devastating effect on the developing brain and often leave MB survivors with severe treatment-related morbidities including neurological, intellectual and physical disabilities [2,3]. Since previous efforts to reduce the morbidity and mortality associated with MB have been restricted by the harsh nature of conventional treatments, there is a clear need for a better treatment options [3,4,5].

MB is classified into four molecular subgroups; wingless (WNT), sonic hedgehog (SHH), Group 3 and Group 4. Groups 3 and 4 tumours are associated with worse clinical outcomes than the other two groups. In particular, Group 3 patients have the lowest survival rate among MB patients and in about 45% of cases already present with metastasis at time of diagnosis [1,6]. MB metastasis occurs through leptomeningeal dissemination where the tumour cells spread through the cerebrospinal fluid to the leptomeninges of the brain and spinal cord [7,8]. Cancer cell invasion and migration play critical roles in metastasis. While cellular transformation, is essential for cancer cell migration and invasion, anchorage-independent growth is the ability of transformed cells to grow and proliferate without the requirement for a substrate and restrictions by cell-ell contacts [9,10,11].

In MB, most available high-throughput drug screens rely on assessing cell proliferation using 2D models. These models, however, fail to address the invasive abilities of cells, that are crucial for MB metastasis and therefore poorly represent tumour cell growth in vivo. Specifically, cell-cell and cell-extracellular matrix interactions, as well as oxygen supply and availability of nutrients are poorly replicated in 2D assays. These limitations likely result in inaccurate selection of drug candidates. Hence, more representative in vitro models of MB are urgently needed that can be used for high-throughput screening of drug candidates.

The soft agar invasion assay is considered a standard method to measure cellular transformation potential in vitro [12]. Cells lacking this ability will die while those with anchorage independence abilities will transform and continue to grow to form colonies of different sizes. The metabolic activity of the formed colonies will be assessed using fluorescent dyes which is a reflective of their direct tumorigenic and invasive potential that is ability to grow without matrix support. This stringency makes the assay a superior tool to test the effectiveness of potential drug candidates [13,14,15,16]. Furthermore, in this assay cells grow as colonies into spheroids that replicates the physiological environment in vivo in terms of cellular interactions and unique growth conditions. Hence, the soft agar invasion assay offers key advantages over conventional 2D assays used for drug screening [17,18,19]. However, standard agar invasion assays are of low-throughput and require manual counting of cell colonies and colony volume. Hence, this approach was so far not suitable for high-throughput drug screens.

This study describes a novel 384-well-based agar invasion assay that assessed the growth of two Group 3 MB cell lines cells in a 3D matrix. Three-dimensional cell growth was quantified by multiplexing two fluorescent substrates that independently quantified growth of viable cells without any detected signal interference. After assay validation using MB-specific chemotherapeutic agents, this assay was successfully used to identify a specific group of Ca^2+^ channels as potential drug targets that could provide an entirely new treatment strategy for MB patients.

## 2. Results

### 2.1. Optimisation of Cell Seeding Density and Culture Time

**Figure 1:**
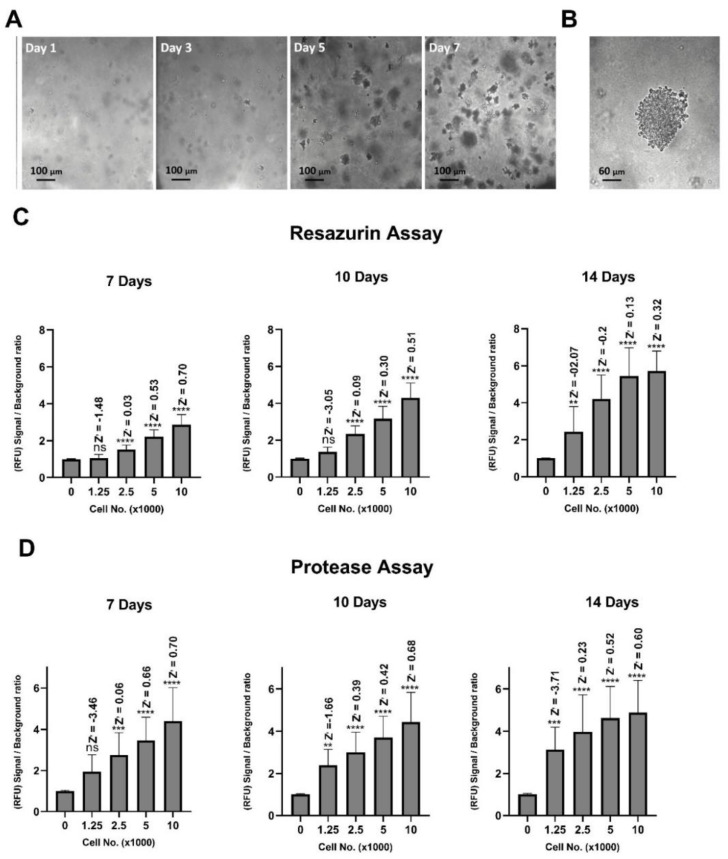
Optimisation of cell density and culture time for D341 cells. Representative images were acquired using an IN Cell Analyzer 2200 including (**A**) colony development over time at 10× magnification and (**B**) a single colony after 7 days incubation at 20× magnification. Quantitative relative fluorescence level of (**C**) resazurin, and (**D**) GF-AFC substrates from cells seeded at four different densities and cultured for 7, 10 and 14 days. Data expressed as mean ± standard deviation from three independent experiments with four replicates each. ns = not significant (*p* > 0.05), ** *p* < 0.01, *** *p* < 0.001, **** *p* < 0.0001 (one-way ANOVA with Dunnett multiple comparisons test compared with the no-cells group). Z’ = Z’-factor.

Optimal cell seeding numbers were chosen based on signal to background fluorescent ratio and Z’-factor values obtained from each of the cell viability assays. Z’-factor is a statistical method used to judge the quality of a given bioassay, and its suitability for high-throughput screening (HTS). Z’-factor values between 0.5 and 1 suggest excellent assay quality, while values of 0.5 or below are predictive of marginal or weak assays for HTS screening purposes [20]. Four different cell numbers (from 1250 to 10,000) were plated in each well of three identical 384-well plates and allowed to grow for 7, 10 and 14 days in soft agar before subsequent analysis. At each respective time point, one of the plates was analysed by each of the two assays individually. Representative images of D341 colonies grown in soft agar over time and higher magnification of one colony at day 7 are shown in Figure 1A,B, respectively. Incubation of D341 cells with resazurin substrate (6 h) or glycyl-phenylalanyl-aminofluorocoumarin (GF-AFC) substrate (indicating protease activity) (3 h), at 10,000 cells/well for 7 days produced a signal to background ratio of 2.87 ± 0.52 (Z’-factor = 0.70) and 4.4 ± 1.55 (Z’-factor = 0.70) for resazurin and protease assays, respectively (Figure 1C,D). For the resazurin assay, although 10 day growth time resulted in a higher signal to background ratio compared to 7 days, a clear reduction in Z’-factor value (0.70 at day 7 compared to 0.51 at day 10) was detected (Figure 1C), rendering the assay less suitable for high-throughput analysis. GF-AFC substrate for the same growth periods showed no improvement in signal to background ratio and a similar trend in reduction of Z’-factor with longer timepoints was observed (Figure 1D). Higher cell densities at the 7-day timepoint were assessed, however signals from both substrates appeared to reach saturation at these higher seeding densities (Supplementary Materials Figure S1). Experiments with another MB cell line, D283, showed similar results compared to D341 cells (Figure S2). Based on these data, a density of 10,000 cells per well with a 7-day culture time was used for future experiments.

### 2.2. Optimisation of Incubation Time with Resazurin and GF-AFC Substrates

**Figure 2:**
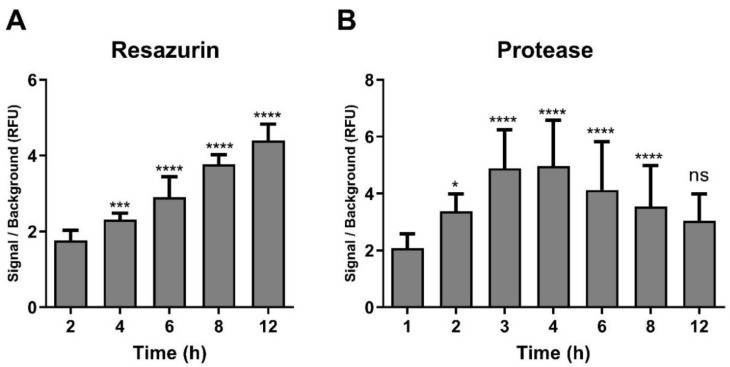
Optimisation of exposure times for the resazurin and GF-AFC substrates. Plates were incubated with (**A**) resazurin, or (**B**) GF-AFC substrates and analysed at different time points. Data are expressed as mean ± standard deviation from three independent assays with four replicates each. ns = not significant (*p* > 0.05), * *p* < 0.05, *** *p* < 0.001, **** *p* < 0.0001 (one-way ANOVA with Dunnett multiple comparisons test compared with the first timepoint group).

Next, incubation times with substrates were optimised. Cells (10,000/well), were incubated with the resazurin substrate for timepoints from 2 to 12 h. A 6 h incubation time resulted in a significant increase in signal to background ratio in D341 (Figure 2A) and D283 (Figure S3A) cells. A further increase of incubation time up to 12 h increased the signal to background ratio. However, this timepoint would be difficult to incorporate into normal lab routines and could also lead to artefacts based on continued cell growth over this time period. For the protease assay, less than 3 h incubation time with the GF-AFC substrate are recommended by the manufacturer [21]. Therefore, different time intervals up to 12 h were tested. The fluorescent signal to background ratio for this assay peaked at 3 h beyond which the fluorescence signal declined, which indicated a gradual loss of signal over time in both D341 (Figure 2B) and D283 (Figure S3B) cells. Based on these results, 6 h and 3 h incubation times for resazurin and protease substrates, respectively, were selected for all further studies.

### 2.3. Multiplex Assay Validation

**Figure 3:**
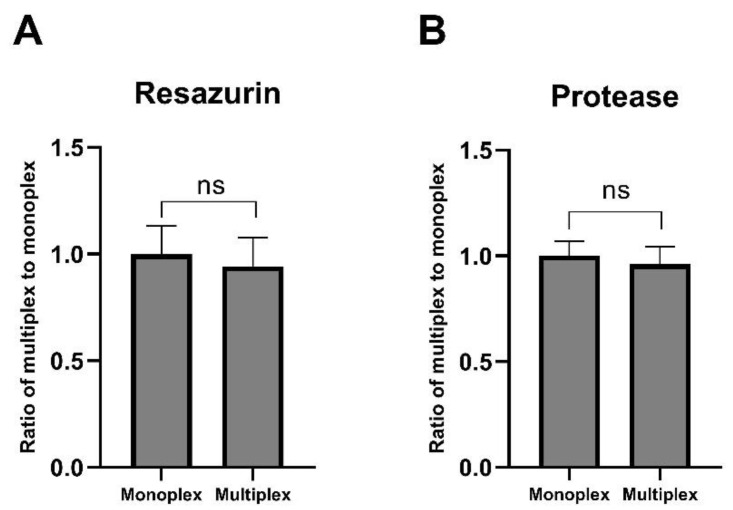
Multiplexing resazurin and protease assays in D341 cells. For the monoplex assay, agar plates were incubated with resazurin substrate (6 h) or GF-AFC substrate (3 h) alone. For the multiplex assay, plates were incubated with resazurin substrate for 3 h followed by GF-AFC substrate in the same well for another 3 h. Plates were analysed for (**A**) resorufin signal at 560/590 nm excitation/emission and (**B**) GF-AFC signal at excitation/emission wavelength of 380/505 nm. Data expressed as mean ± standard deviation from three independent experiments with four replicates each. ns = not significant (*p* > 0.05), *t*-test with two-tailed comparison.

For ease of assay performance, to save time and as a way of data normalisation, the resazurin and protease assays were tested to be multiplexed. For this purpose, cells were incubated with resazurin (6 h) and GF-AFC substrate (3 h) alone or in combination. In both D341 cells (Figure 3) and D283 cells (Figure S4), multiplexing both substrates did not result in a significant change to each signal when compared to each substrate added alone (monoplex). This suggested that these assays have compatible chemistries and without signal interference, and therefore can be used together.

### 2.4. Assessment of Signal Distribution across a Well

**Figure 4:**
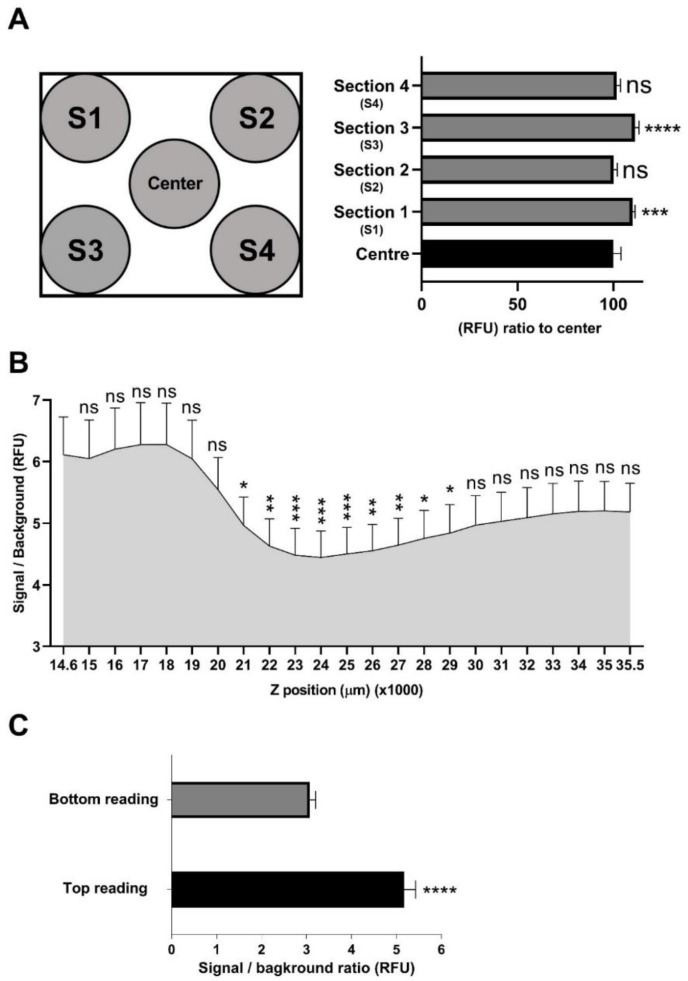
Signal distribution across a well using resazurin substrate. Data expressed as mean ± standard deviation from three independent assays with four replicates each. (**A**) Measurements taken from different sections within one Z-position inside a single well (multiple read, 2 × 2 with 250 µm distance from border of the read regions) using Tecan Spark 20 M multimode microplate reader: ns = not significant (*p* > 0.05), *** *p* < 0.001, **** *p* < 0.0001, one-way ANOVA with Dunnett multiple comparisons test compared to reading from the centre. (**B**) Measurement at different Z-positions within a well ranging from 14600–35500 µm: ns = not significant (*p* > 0.05), * *p* < 0.05, ** *p* < 0.01, *** *p* < 0.001, one-way ANOVA with Dunnett multiple comparisons test compared to reading at Z-position of 14,600 μm. (**C**) Top versus bottom reading: **** *p* < 0.0001, *t*-test with two-tailed comparison test.

Next, the homogeneity of the fluorescence signal from each dye across individual wells was measured to identify possible sources of artefacts. Uneven distribution of the fluorescence signal can reduce assay reliability and is dependent on the fluorescence plate reader used. In particular, the position of the reading head (in x, y and z direction, top or bottom detection) and size of the measurement area within each well can affect assay performance. Therefore, cells incubated with resazurin and GF-AFC substrates were read using different Z-positions, different areas within one Z-position and bottom- versus top-detection modalities. For the resazurin substrate, reading wells from five different sections within one Z-position including four corners and the centre sections did not show a considerable difference in fluorescence signal in between sections in D341 cells, although a modest increase was observed in Section 1 and Section 3 (Figure 4A). GF-AFC substrate also showed a quite homogeneous signals among different sections with a modest increase in Section 2 and Section 4 (Figure S5A). Wells were also scanned from different Z-positions across a well, which showed a drop in signal at Z-positions between 21,000 μm to 29,000 μm compared to the top Z-position at 14,600 μm (Figure 4B). Furthermore, top reading demonstrated a significantly higher signal to background ratio compared to bottom reading in both D341 cells (Figure 4C) and D283 cells (Figure S5B). Collectively, these data suggest that both resazurin and GF-AFC substrates produce almost homogeneous signals across one plane and higher signals closer to the top of a well. Based on these results, top reading is recommended for this assay, which was also used for all subsequent studies at a Z-position of 18,000 μm.

### 2.5. Assessment of Vincristine and Lomustine Toxicity

**Figure 5:**
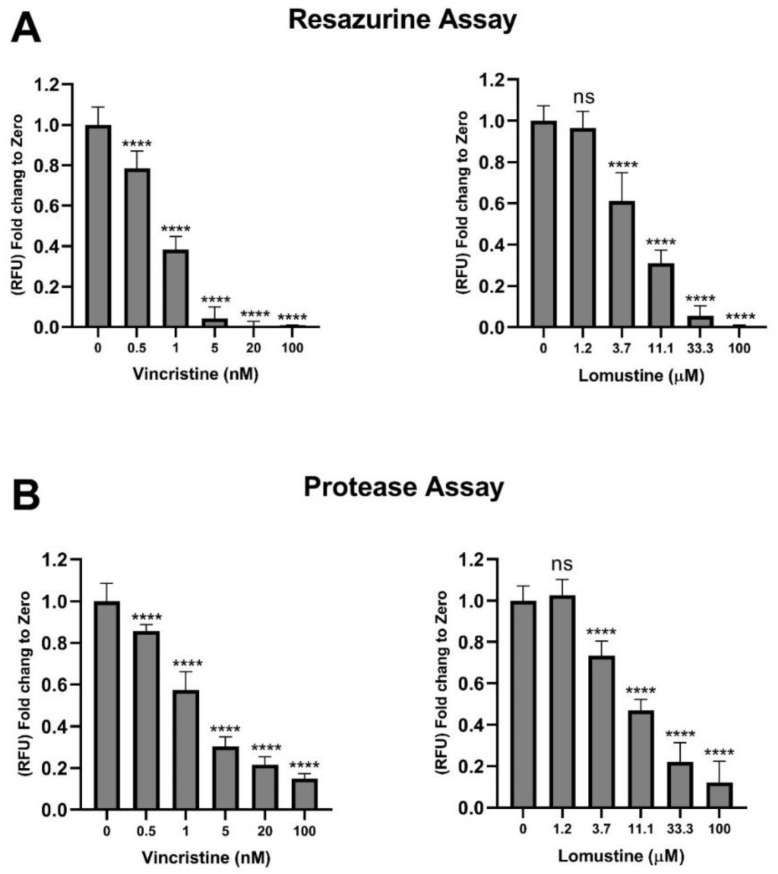
Cytostatic effect of vincristine and lomustine on medulloblastoma (MB) cells. D341 cells (10,000 cells/well in agar) were treated with different concentrations of vincristine (0–100 nM) or lomustine (0–100 µM). After 7 days, plates were analysed using the established multiplex assay and read using plate reader for (**A**) resazurin and (**B**) GF-AFC substrates. Data expressed as mean ± standard deviation from three independent assays with four replicates each. ns = not significant (*p* > 0.05), **** *p* < 0.0001 (one-way ANOVA with Dunnett multiple comparisons test compared with the non-treated 0 control group).

To evaluate the sensitivity of the assay, the effect of two commonly used chemotherapeutic agents clinically used for the treatment of MB, vincristine and lomustine, were tested in the multiplex agar assay. D341 cells embedded in agar were treated with vincristine (0.5–100 nM) or lomustine (1.2–100 µM) for 7 days. At day 7, plates were analysed as described above. Both agents, concentration dependently suppressed the growth of cells in the agar matrix as indicated by a reduction in fluorescence signal from resazurin (Figure 5A) and GF-AFC (Figure 5B). These results further validate and confirm the sensitivity of the multiplex assay.

### 2.6. Assessment of Plasma Membrane Calcium Channel Modulators

**Figure 6:**
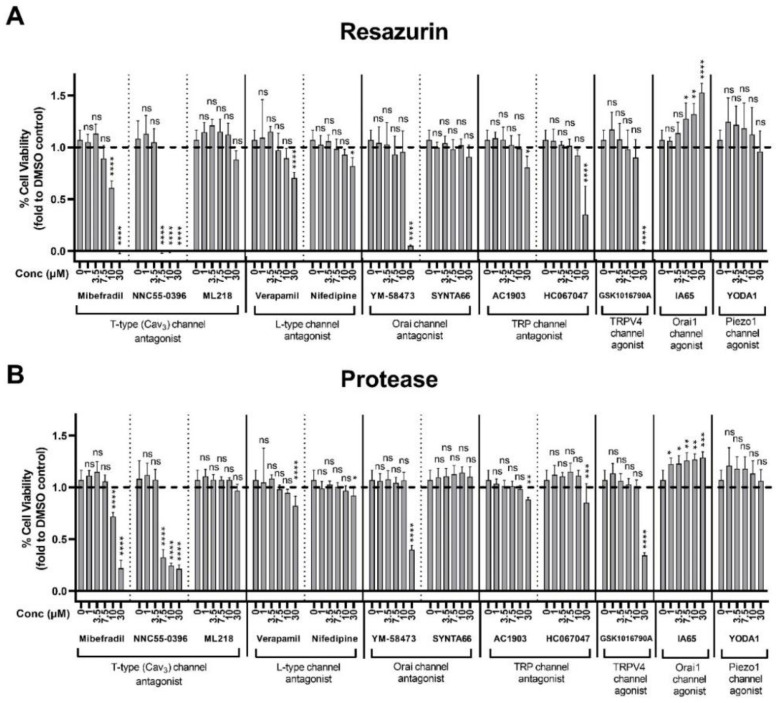
Effect of plasma membrane calcium channel modulators on substrate-independent growth of MB cells. D341 cells (10,000 cells/well in agar) were treated with DMSO (solvent control) or different concentrations of test compounds from 1 to 30 µM. After 7 days, plates were analysed by quantifying (**A**) resazurin and (**B**) GF-AFC substrate fluorescence. Data represent mean ± standard deviation from two independent assays with four replicates each. The control group for both assays is the same and 0.3% DMSO was used as solvent control for all the compounds except for NNC55-0396, where media was used. ns = not significant (*p* > 0.05), * *p* < 0.05, ** *p* < 0.01, *** *p* < 0.001, **** *p* < 0.001 (one-way ANOVA with Dunnett multiple comparisons test compared with the 0-control group).

This assay was next used to screen a panel of pharmacological modulators of plasma membrane calcium channels for their effects on the substrate-independent growth of D341 cells. A panel of calcium channel agonists and antagonists targeting different classes of channels involved in Ca^2+^ influx were used that included ORAI channels, transient receptor potential (TRP) channels, mechanosensitive PIEZO channels, L-type and T-type voltage-gated Ca^2+^ channels. Among these compounds, inhibitors of the T-type Ca^2+^ channels, mibefradil and NNC55-0396 decreased cell growth in a clear concentration-dependent manner indicated by a significant reduction in the resazurin and GF-AFC fluorescence signals (Figure 6A,B). In contrast, activation of the ORAI1 channel using IA65 increased cell growth in a concentration dependent manner, while inhibition of this channel with YM-58473 at the highest concentration (30 µM) inhibited cell growth (Figure 6A,B).

## 3. Discussion

This study describes a novel high-throughput assay to assess colony formation and growth of medulloblastoma cells in an agar matrix using two fluorescent substrates measuring cell growth and viability via distinct mechanisms. The use of agar instead of collagen makes this assay affordable and compatible with fluorescence measurements using standard plate readers. In addition, this assay confers several advantageous compared to conventional 2D assays: it better represents tumour mass in vivo in terms of cell-cell interaction and growth of cells into spheroids; it also better represents cells with invasive capacities given that these cells can better proliferate into the agar matrix. Indeed, soft agar colony formation assay is one of the most rigorous assays available to assess cell invasion [12]. While similar high-throughput assays have been optimised for other cancer cells, this has not been the case for medulloblastoma, a cancer that shows a high level of metastasis [8], and lacks optimised 3D high-throughput assays for drug screening.

This assay was optimised for 384-well plate format, and could likely be adapted to 96-well format, as well as to 1536-well format with the aid of a robotic platform for liquid handling. The results of this study demonstrate that two fluorescent substrates can be multiplexed for ease of assay performance, to save time and as a way of data normalisation. Given the distinct emission and excitation wavelengths of the two substrates used in this assay (resazurin and GF-AFC), no signal interference was observed and to our knowledge, this is the first time that these two substrates have been multiplexed in a high-throughput assay.

The use of two independent mechanisms to assess cell viability reduces artefacts that may be associated with drug-substrate interactions. For instance, resazurin can be readily reduced to resorufin in the presence of interfering chemical species without the presence of any cellular activity, which is of particular relevance for compounds with thiol and carboxylic acid moieties [22]. The presence of another independent readout helps to avoid artefacts and therefore any misinterpretation of results. Furthermore, resazurin, in addition to indicating cell viability, is frequently used to assess cell metabolic activities given the reduction of resazurin to resorufin by electron transfer from NADPH [22]. Hence, this assay can also be used to screen for drug effects on cell metabolism. In this case, data from the protease assay may be used to normalise data from the resazurin assay given that this assay measures constitutive protease enzyme activity that is based on fixed enzyme quantity in each cell and is directly correlated to cell number.

A previous study reported the use of imaging-based system for HTS against cell invasion in agar [23]. In comparison to our approach, use of imaging system to study colony formation in agar is more labour intensive and time consuming which will undermine the purpose of a high throughput screen. Furthermore, in imaging-based screen the thickness of the agar colonies containing layer is often lower than conventional agar test to allow for better quality imaging. The effect of this on the performance of the test needs to be further studied to ensure assay suitability. Finally, in our assay, we employed two dyes instead of one which will reduce the possibility of false negative response that might result from dye-molecule interaction.

Optimisation of cell density and substrate incubation time in this assay resulted in Z´-factor values of above 0.6, further supporting the suitability of this assay for high-throughput screening. This assay also provided appropriate signal to background ratios of above 3 and 5 for resazurin and the protease assay, respectively, indicative of the strength and accuracy of the assay [20]. The described assay is characterized by homogeneous fluorescent signal across one focal plane, which making this assay suitable for different plate readers and reading formats. Nevertheless, higher signal levels were obtained from top reading compared to bottom reading, which as to be taken into consideration and are likely the consequence of higher colony numbers closer to the top of the agar.

This study also used this new assay to screen a panel of plasma membrane calcium channel modulators. Calcium signalling critically affects many of the hallmarks of cancer including cell proliferation, apoptosis resistance, metastasis and angiogenesis [24,25,26]. Indeed, altered expression and/or activity of plasma membrane calcium channels have been reported in several types of cancers [24,27], and pharmacological modulators of specific Ca^2+^ influx channels have been proposed to represent promising future agents for cancer therapy [28,29,30]. Our screen showed that inhibition of T-type Ca^2+^ channels with mibefradil and NNC55-0396 effectively inhibited the growth of MB cells, in contrast to ML218 another inhibitor of the T-type channels that did not significantly suppress cell growth. Although we cannot rule out off target effects of these inhibitors, this discrepancy likely represents different inhibitors’ selectivities towards T-type calcium channel subtypes: mibefradil, a broad T-type calcium channel blocker and weak L-type calcium channel blocker [31], NNC55-0396, a CaV3.1 and CaV3.2 blocker [32,33], and ML218, a CaV3.2 and CaV3.3 blocker [34]. Previously, inhibition of CaV3 channels with mibefradil was reported to suppress the growth and stemness of glioblastoma stem-like cells and sensitise them to temozolomide chemotherapy [35]. Mibefradil is a commercially available selective inhibitor of CaV3, originally approved for treatment of hypertension and angina pectoris. This drug was later withdrawn from market due to interactions with other drugs frequently administered to patients with cardiovascular diseases. However, mibefradil recently successfully completed a Phase I trial for recurrent high-grade glioma, and in combination with temozolomide was well-tolerated in patients with no toxicity (ClinicalTrials.gov identifier NCT01480050 and publication [36]). In comparison to mibefradil, its analogue NNC55-0396 has higher blood-brain-barrier permeability [32,37] and shows lower non-specific inhibition of L-type calcium channels [32,37], as well as cytochrome P450 [37] compared to mibefradil. The results of the present study also showed that activation of the ORAI1 channel with IA65 [38], enhanced the growth of MB cells while its inhibition with YM-58473 at 30 µM reduced cell growth. ORAI channels are involved in store-operated calcium entry that is initiated by the depletion of endoplasmic reticulum Ca^2+^ stores [39]. ORAI channels have been shown to regulate metastasis-related processes in several cancers including breast [40,41], gastric [42] and glioblastoma [43]. Based on these results, further studies are required to assess the detailed roles of CaV and ORAI channels in MB cell invasion and other metastasis-related processes.

## 4. Materials and Methods

### 4.1. Chemicals and Reagents

Dimethylsulfoxide (DMSO), Minimum Essential Medium Eagle (EMEM, M0643), sodium bicarbonate (NaHCO3), noble agar (A5431), resazurin sodium salt (199303), Vincristine (V0400000), Lomustine (L5918), YM-58483 (Y4895), Synta66 (SML1949), GSK1016790A (G0798), HC-067047 (SML0143), AC1903 (SML2244), ML218 (SML0385), Mibefradil dihydrochloride hydrate (M5441), NNC55-0396 hydrate (N0287), Yoda1 (SML1558), Verapamil hydrochloride (V4629) and Nifedipine (N7634) were purchased from Sigma-Aldrich (Ryde, NSW, Australia). IA65 was a kind gift from Professor William Denny and Dr. Ralph Stevenson, and was synthesised as previously described [28]. CellTiter-Fluor™ Cell Viability (G6081) was obtained from Promega (Madison, WA, USA).

### 4.2. Cell Culture

D283 (HTB-185™) and D341 (HTB-187™) were purchased from The American Type Culture Collection (ATCC, Manassas, VA, USA). Cells were cultured in EMEM supplemented with 10% and 20% fetal bovine serum (FBS) respectively followed by the supplier recommendation. Cells were maintained at 5% CO_2_ and 37 °C inside a humidified incubator and passaged twice a week.

### 4.3. Preparation of Agar Layers

Agar mixture was prepared as a 5% solution by adding 5 g of noble agar to 100 mL of distilled water. Agar solution was then autoclaved for melting into a homogenous solution and for sterilization. The mixture can be made in advance and stored at 4 °C; however, it should be melted again using microwave oven at the time of the experiment until agar solution is completely homogenous. The prepared solution was used up to 3–4 times before preparing fresh mixture.

For preparation of agar bottom layer, a 0.6% agar solution was prepared by adding 1.2 mL of 5% agar solution (65 °C) to 8.8 mL of the EMEM media preheated to 40 °C using water bath. Subsequently, 10 µL/well of 0.6% agar solution was added to 384-well plates (781091, µClear, Greiner, NSW, Australia). Immediately after adding the bottom layer, the plate was gently tapped multiple times against the surface of the cell culture hood to ensure that the agar layer covers the entire bottom of all wells. Plates were then kept at room temperature for 1 h to allow the bottom agar layer to solidify. During the one-hour incubation, cells to be used in the assay were prepared. Cells were first suspended into a single cell suspension, counted and kept at 39 °C water bath for 5 min. Agar top layer was prepared by mixing 1.6 mL of 5% agar medium (around 42 °C) with 18.4 mL of cell suspension (kept at 39 °C for 5 min). A total of 50 µL of cell-agar suspension was then quickly plated into each well producing a top layer of 0.4% agar medium. The plate was tapped as described above and kept at room temperature for 1 h before transferring it to the incubator.

### 4.4. Cell Viability Measurement

Cell viability was quantified as a surrogate marker of cell invasion potential. by multiplexing two cell viability assays, resazurin and CellTiter-Fluor™. Resazurin is used as a fluorometric cell viability assay that measures cells viability and metabolic activity [44]. The non-fluorescent resazurin is reduced via mitochondrial reductase enzymes of live cells to the highly fluorescent product resorufin. The amount of resorufin is quantified using a microplate fluorometer with 560/590 nm excitation/emission filters. The signal magnitude is directly proportional to the number of metabolically active cells. A 4.4 mM resazurin stock solution was prepared by dissolving 11 mg of resazurin salt in 10 mL of phosphate-buffered saline (PBS) solution. This solution is aliquoted and stored at −20 °C for later use but no more than 3 months. A working solution of 440 µM was freshly prepared for each assay by diluting resazurin stock solution ten times in culture medium as recommended [44,45].

CellTiter-Fluor™ Cell Viability Assay is a cell viability assay that measures constitutive live cell protease enzyme. The assay principle is based on the cleavage of cell permeable substrate GF-AFC by cell proteases. Cleavage of the substrate generates a fluorescent signal proportional to the number of living cells. Cells with compromised cell membrane lose this protease activity due to enzymes leaking out and are unable to metabolise the substrate [36]. Working solution for the CellTiter-Fluor^™^ Assay was prepared according to the manufacturer’s recommendation. Briefly, 10 μL of the GF-AFC substrate was dissolved in 2 mL of assay buffer. This solution was used in conjugation with the resazurin assay as discussed below [21,46].

Both assays were multiplexed by adding assay solutions sequentially in the same wells. Briefly, media was removed from agar plates by gently tapping the plate upside down on a paper towel. Subsequently, 7.5 µL of the 440 µM resazurin working solution was added to each well and incubated for 3 h at 37 °C. This was followed by addition of GF-AFC substrate (7.5 µL/well), prepared as mentioned above, without removal of the resazurin solution, to the same wells and a further 3 h incubation at 37 °C. After this, the plate was removed from the incubator and analysed using a fluorescence plate reader (Tecan Spark 20M, multimode microplate reader) at 560/590 nm excitation/emission for resazurin and 380/505 nm excitation/emission for the GF-AFC substrate.

### 4.5. Addition of Test Compounds

Test and reference compounds were reconstituted in DMSO except for NNC55-0396, which was reconstituted in water. Stock solutions were prepared as single use aliquots and stored at -20 °C until used. Working solutions were prepared by further diluting test compounds to 10 mM in DMSO (NNC55-0396 was diluted in water). The 1:5 serial dilutions were prepared in culture media (5–150 µM). Post plating (24 h), 15 µL of each compound (or DMSO control) was transferred from the drug dilution plate to the cell plate using an 8-channel pipettor. D341 cells embedded in agar were treated with test compounds for 7 days. At day 7, plates were analysed using the multiplex resazurin-protease assay as described above.

### 4.6. Calculation of Signal to Background Ratio and Z’-factor

Signal to background ratio and Z’-factor were used to assess the suitability of the developed assay for high-throughput screening. Signal to background ratio was calculated by dividing the average value of the signal from wells containing cells by the average value from control wells with no cells. Z’-factor, a measure of statistical effect size, is a simple parameter that is widely used to assess the performance and the reproducibility of high-throughput assays. Z’-factor was calculated based on the below equation as previously described [18].

(1) $$Z´=1-\frac{3\;\times \mathrm{SD}\;\mathrm{of}\;\mathrm{sample}+3\;\times \mathrm{SD}\;\mathrm{of}\;\mathrm{control}}{\mathrm{mean}\;\mathrm{of}\;\mathrm{sample}-\mathrm{mean}\;\mathrm{of}\;\mathrm{control}}$$

Z’-factor of 1 indicates an ideal assay for drug screening. A value between 0.5 and 1 suggests an excellent assay quality, while a value of 0.5 or less is a predictive of a marginal or a weak assay for screening purposes [18]. Control wells for Z´-factor calculation were prepared by adding detergent (15 μL of 0.5% triton in PBS, filter sterilized) to produce minimal signal [14,18], 24 h after seeding.

### 4.7. Statistical Analysis

Statistical analysis was performed using GraphPad Prism version 8.2.1 (San Diego, CA, USA). Specific statistical tests used are described in each Figure legend. Data are presented as mean ± standard deviation.

## 5. Conclusions

In conclusion, this study described a 3D 384-well format soft agar colony formation assay that is used in conjunction with standard fluorescence plate readers. It was developed for Group 3 medulloblastoma cells but could easily be translated to suit tumour cells of different origins. This HTS assay can be used to screen for agents that affect medulloblastoma cell growth in a matrix that mimics 3-dimensional tumour growth in vivo.

## Supplementary Materials

The following are available online at https://www.mdpi.com/1424-8247/13/11/368/s1, Figure S1: Optimisation of cell density. Cell densities of up to 20,000 cells/well of the D341 cell line were tested. Data represents quantitative relative fluorescence levels of (A) resazurin, and (B) GF-AFC substrate from cells seeded at six different densities and cultured for 7 days, Figure S2: Optimisation of cell density and culture time. Data represent quantitative relative fluorescence level of (A) resazurin, and (B) GF-AFC substrates from D283 group-3 MB cells seeded at four different densities and cultured for 7, 10 and 14 days, Figure S3: Optimisation of exposure time. D283 cells were incubated with (A) resazurin, or (B) GF-AFC substrates and analysed at different time points, Figure S4: Multiplexing resazurin and protease assays. For monoplex detection, D283 cells were incubated with resazurin (6 h) or GF-AFC (3 h) alone. For multiplex detection, cells were incubated with resazurin for 3 h followed by GF-AFC for another 3 h. Plates were analysed for the (A) resorufin signal at 560/590 nm excitation/emission and for the (B) GF-AFC signal at excitation/emission wavelength of 380/505 nm, Figure S5: Signal distribution across a well using GF-AFC substrate in D341 MB cells. Data are expressed as mean ± standard deviation from three independent assays with four replicates each. (A) Measurements taken from different sections within one Z-position inside a single, (B) Top versus bottom reading.
