# Supplementary material for: Development of a High-throughput Agar Colony Formation Assay to Identify Drug Candidates against Medulloblastoma
Source: Pharmaceuticals (Basel). 2020 Nov 5;13(11):368. doi: 10.3390/ph13110368 (PMC7694510; doi:10.3390/ph13110368)
Supplement: Supplementary file 1 [file pharmaceuticals-13-00368-s001.pdf]

Development of a high-throughput agar colony formation assay to identify drug candidates against medulloblastoma

Mohammed Sedeeq, Ahmed Maklad, Nuri Gueven, Iman Azimi

Supplementary material

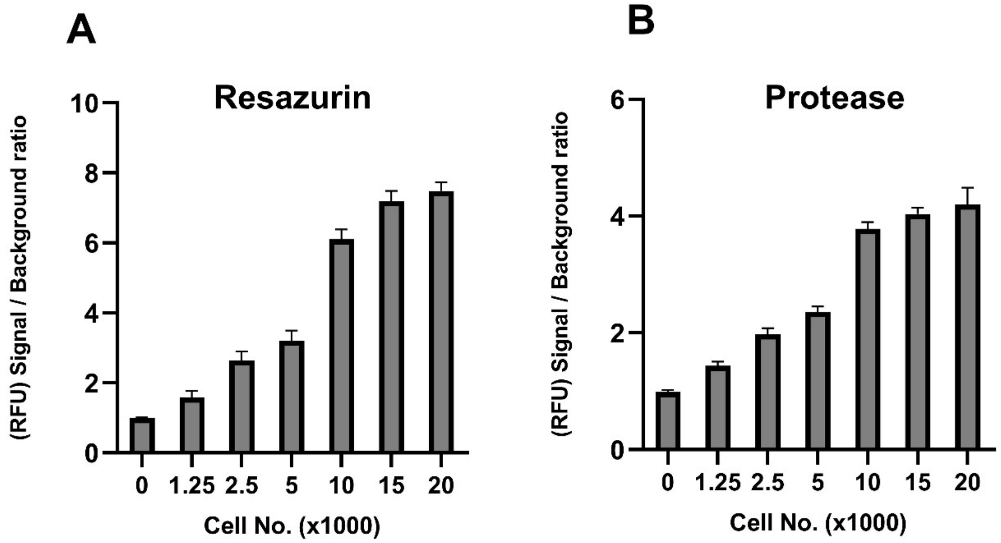

Figure S1. **Optimisation of cell density.** Cell densities of up to 20, 000 cell/well of the D341 cell line were tested. Data represents quantitative relative fluorescence levels of (**A**) resazurin, and (**B**) GF-AFC substrate from cells seeded at six different densities and cultured for 7 days.

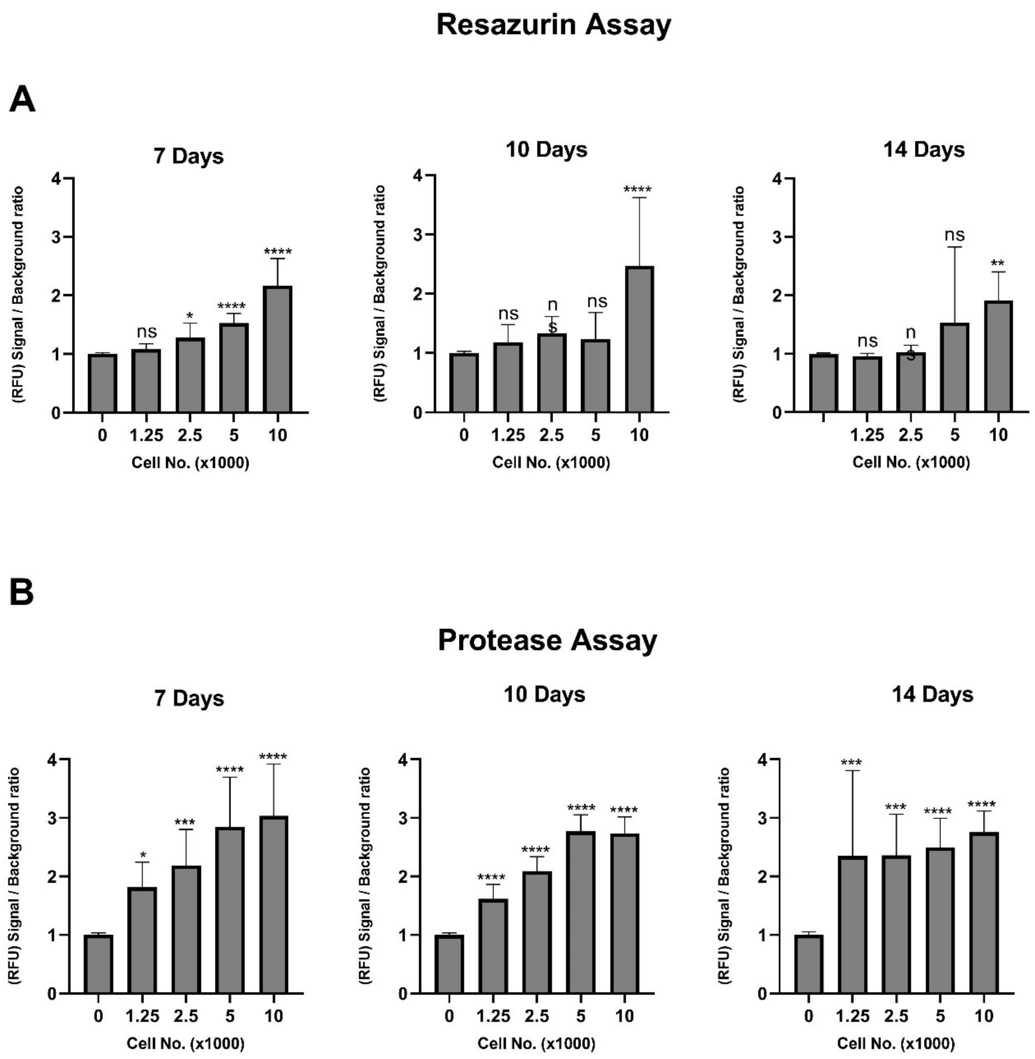

Figure S2. **Optimisation of cell density and culture time.** Data represent quantitative relative fluorescence level of (**A**) resazurin, and (**B**) GF-AFC substrates from D283 group-3 MB cells seeded at four different densities and cultured for 7, 10 and 14 days. Data are expressed as mean ± standard deviation from three independent experiments with four replicates each. ns = not significant(p>0.05), ***p<0.001, ****p<0.0001 (one-way ANOVA with Dunnett multiple comparisons test compared with the 1250 cells group.

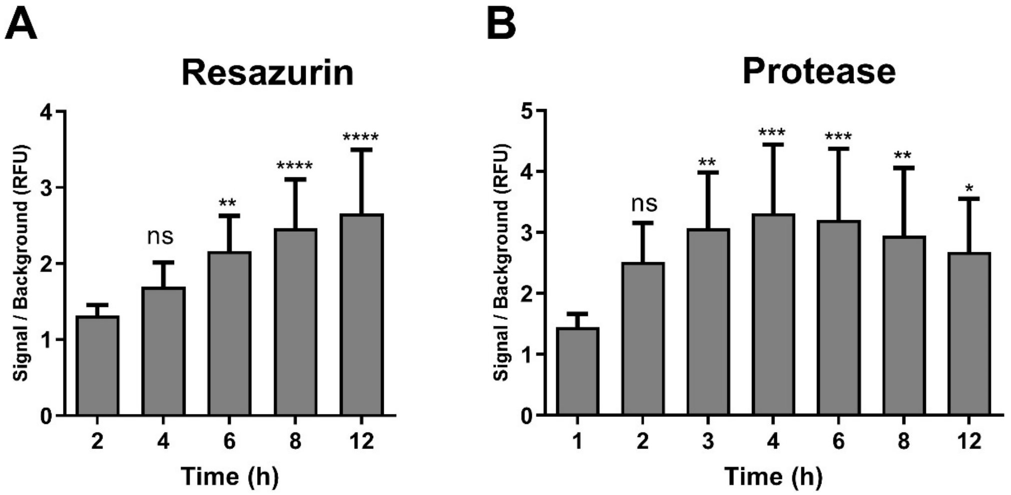

Figure S3. **Optimisation of exposure time.** D283 cells were incubated with (**A**) resazurin, or (**B**) GF-AFC substrates and analysed at different time points. Data are expressed as mean ± standard deviation from three assays with four replicates each. ns = not significant ($p>0.05$), *$p<0.05$, **$p<0.01$, ***$p<0.001$, **** $p<0.0001$ (one-way ANOVA with Dunnett multiple comparisons test compared with the first timepoint group).

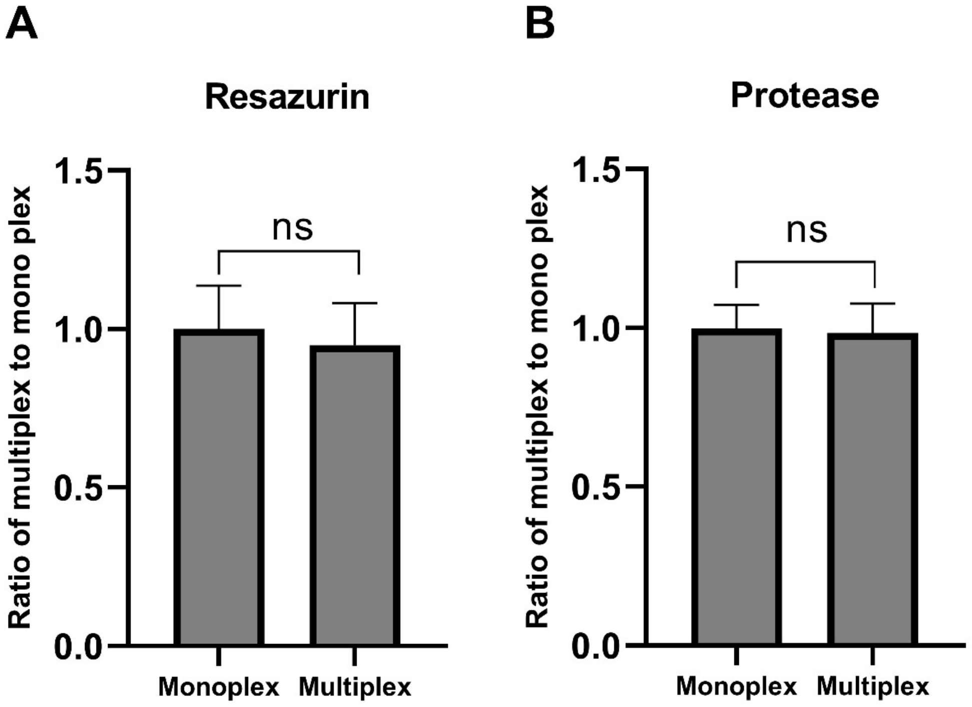

Figure S4. **Multiplexing resazurin and protease assays.** For monoplex detection, D283 cells were incubated with resazurin (6 h) or GF-AFC (3 h) alone. For multiplex detection, cells were incubated with resazurin for 3 h followed by GF-AFC for another 3 h. Plates were analysed for the (**A**) resorufin signal at 560/590 nm excitation/emission and for the (**B**) GF-AFC signal at excitation/emission wavelength of 380/505 nm. Data are expressed as mean ± standard deviation from three independent assays with four replicates each. ns = not significant, (p>0.05), t-test with two-tailed comparison.

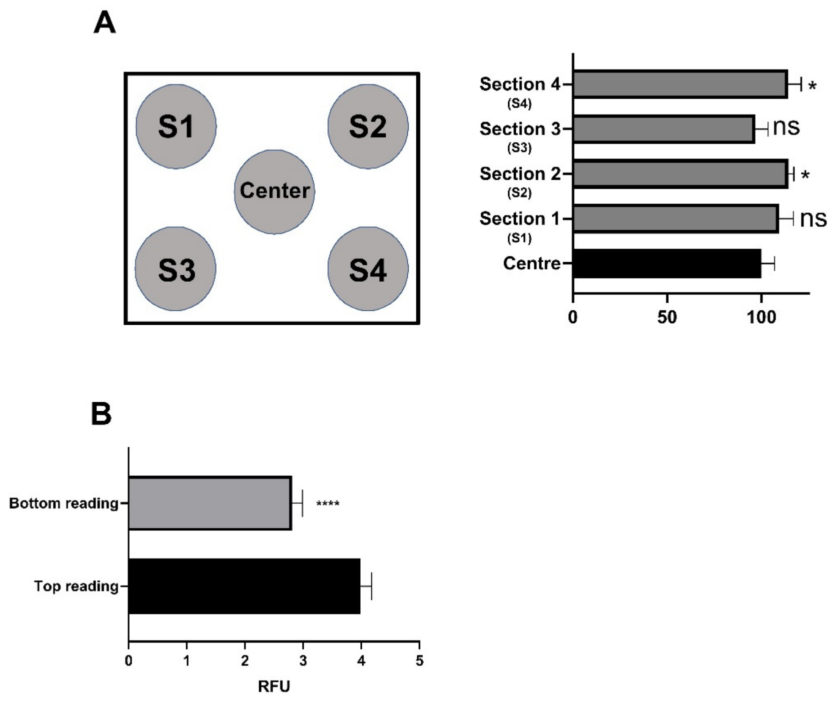

Figure S5. Signal distribution across a well using GF-AFC substrate in D341 MB cells. Data are expressed as mean ± standard deviation from three independent assays with four replicates each. (**A**) Measurements taken from different sections within one Z-position inside a single well (multiple read, 2 X 2 with 250 µm distance from border of the read regions) using Tecan Spark 20M Multimode Microplate Reader, one-way ANOVA with Dunnett multiple comparisons test compared to reading from the center: ns = not significant ($p>0.05$), *$p<0.05$ (**B**) Top versus bottom reading. with two-tailed comparison test compared to reading from bottom side of the well: ****$p<0.0001$.
